# Parent, child, and situational factors associated with parenting stress: a systematic review

**DOI:** 10.1007/s00787-022-02027-1

**Published:** 2022-07-25

**Authors:** Yuan Fang, Jie Luo, Marloes Boele, Dafna Windhorst, Amy van Grieken, Hein Raat

**Affiliations:** 1 Department of Public Health, Erasmus University Medical Centre, Rotterdam, PO Box 2040, 3000 CA Rotterdam, The Netherlands; 2 Department of Pulmonary Department, Franciscus Hospital, Rotterdam, The Netherlands; 3 Department of Cognitive Neuroscience, Donders Institute for Brain, Cognition and Behavior, Radboud University Medical Center, Nijmegen, The Netherlands; 4 TNO Child Health, Leiden, The Netherlands

**Keywords:** Parenting stress, Mothers, Fathers, Risk and protective factors, Social support, Child health

## Abstract

Parenting stress has been related to adverse health outcomes in parents, children, and their families. This systematic review aimed to provide an overview of parental, child, and situational factors related to parenting stress in mothers and fathers. We searched Embase, Medline Epub (Ovid), PsychInfo (Ovid), Web of Science, and Google scholar for studies published between January 1980 and May 2021 evaluating the association between at least one factor and parenting stress. Studies were included only if they reported the association in a general population sample of mothers and fathers with children aged 0–12 years. The parent–child relationship model by Abidin guided the data synthesis. Quality of the evidence was assessed using the Standard Quality Assessment Criteria for Evaluating Primary Research Papers from a variety of fields. In total, 29 studies were included with excellent quality (55%), good (31%), and adequate (14%) methodological quality. There was evidence of an association between maternal depression, child overall problems, child externalizing and internalizing problems, social support, maternal educational level and maternal parenting stress. Evidence was inconsistent for an association between maternal anxiety, family income and maternal parenting stress. There was no evidence of an association for maternal age, child sex and maternal parenting stress. Several modifiable factors (i.e., parental depression and social support) were identified that might guide the development of preventive interventions. Future research should employ longitudinal study designs evaluating protective and risk factors and the pathways that lead to parenting stress, among both fathers and mothers.

**Supplementary Information:**

The online version contains supplementary material available at 10.1007/s00787-022-02027-1.

## Introduction

Parenting stress is the experience of distress or discomfort that results from demands associated with the role of parenting [1, 2]. Parenting stress is common among parents [3–5]. It is estimated that 36–50% of parents experience some levels of parenting concerns about parenting, child behavior or child development [3, 4]. Parents can feel the need to seek professional advice and help [4, 5].

Parenting stress may have a broad impact on parents, children, and may also impact the parent–child relationship [6]. Higher levels of parenting stress have been linked to increased depression, anxiety, and fatigue in parents [7]. Besides, parents who reported higher levels of parenting stress are also likely to have a lower quality of parenting behavior [7, 8]. Parenting stress has also been linked to a number of adverse outcomes of children (e.g., increased emotional and behavioral problems, socio-emotional dysfunction, and lower social competence), either directly, or indirectly via its impact on parents [7–9]. Identifying modifiable factors that relate to parenting stress could benefit health professionals to support parents and children.

Research on the etiology of parenting stress has explored diverse factors and findings showed mixed associations. For instance, while De Stasio [10] and Schellinger [11] observed a negative association between higher levels of social support and parenting stress; a null association was reported by Seah [12] and Mulsow [13]. Moreover, most of the relevant studies were performed among clinical populations (e.g., parents or children suffering from a specific disease or impairment) [14–16], were focused predominantly on mothers, or did not examined differences in associations for mothers and fathers [15, 16]. The factors associated with parenting stress, however, might differ among parents parenting a child with an atypical versus typical development [9, 14, 17], and between mothers and fathers [1, 18].

**Fig. 1:**
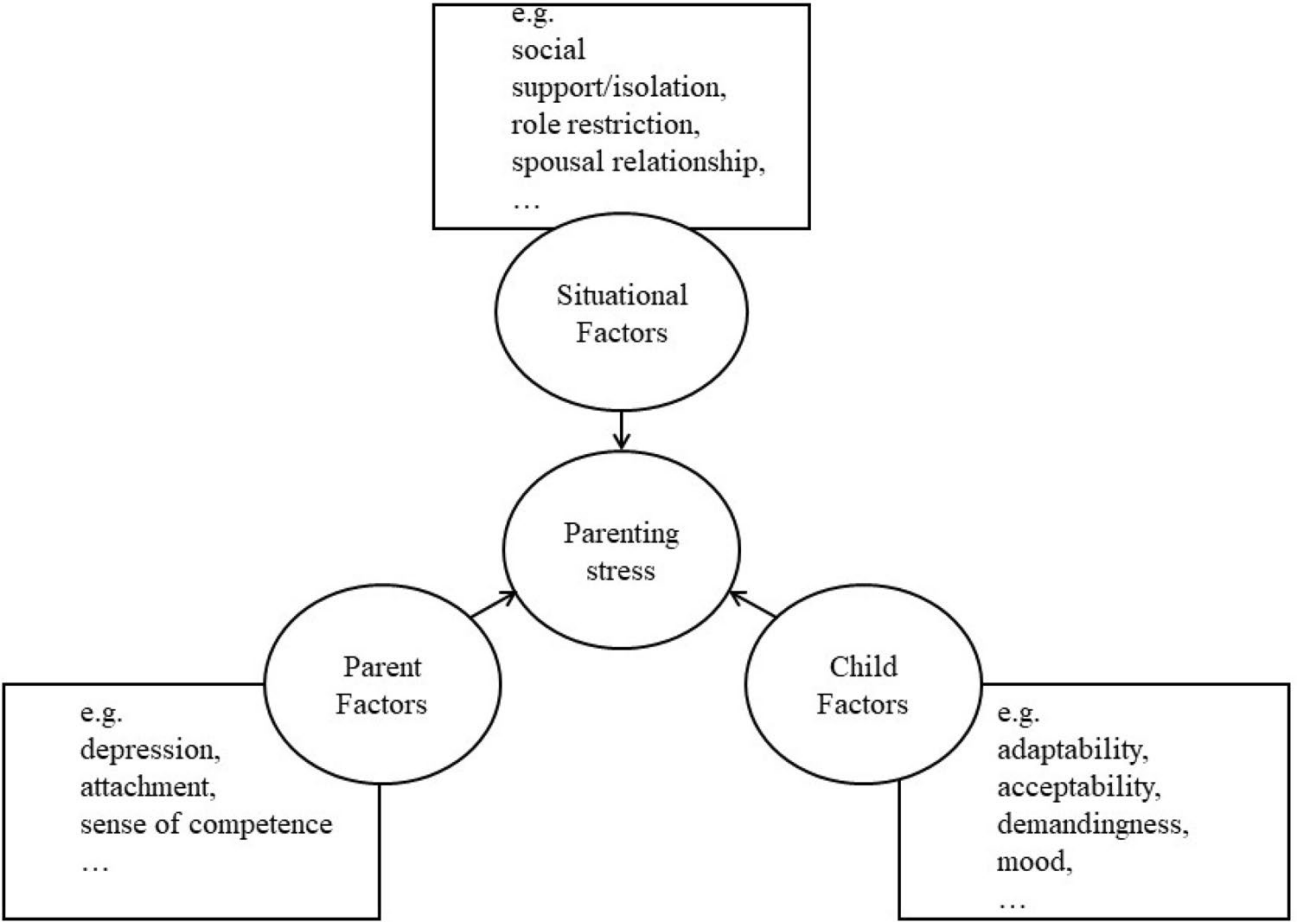
Model of parenting stress. Adapted from Abidin [2, 20]

According to Folkman and Lazarus’ general model of stress [19], stress is the result of the interaction of the stressors, cognitive appraisal, and coping. Stress is when an individual deems that the situation has exceeded his/her resources engaging coping mechanisms to restore functioning. Building on this theory [19], Abidin proposed a comprehensive framework (i.e., the parent–child relationship model) for parenting stress [20]. Although originated already in the 1980s, the framework continues to dominate the literature [21]. Figure 1 illustrated the model that guided this study. Factors associated with total parenting stress were organized into three domains (i.e., parent, child and situational), following Abidin et al. [2, 20, 21]. The parent domain involves aspects of parental functioning and personality components, such as depression, attachment, and sense of competence. The child domain refers to a child’s temperamental and behavioral factors (i.e., adaptability, acceptability, demandingness, mood, and distractibility/hyperactivity. And the situational domain includes social support/isolation, role restriction and spousal relationship.

The parenting stress index (PSI), first developed by Abidin, is an established measure to access perceived parenting stress in caregivers. It is a self-report instrument for parents with reliable psychometric properties, validated across cultures [14]. This instrument has been widely used by clinicians and researchers in screening, diagnostic assessment, and measurement of intervention [22, 23]. The PSI calculates scores on two domains of parenting stress: child and parent. The total score over both domains provides an overall indication of parenting stress that a parent experiences [20, 23].

## The review

### Aims

The current systematic review provides an overview of studies evaluating parent, child, and situational factors associated with parenting stress in both mothers and fathers of children age 0–12 years old in the general population. Parenting stress levels fluctuate according to the developmental stages [24, 25]. We focused on children aged 0–12 years old, as these children spend the most time with their parents and frequently interact with their parents [25]. Parents still play an important role in shaping children’s lives and everyday experiences and vice-versa [26, 27].

### Designs

#### Registration

This systematic review followed the Preferred Reporting Items for Systematic Reviews and Meta-Analysis (PRISMA) guidelines (available in the supplemental materials) [28]. The systematic review protocol was registered at PROSPERO (registration number: CRD42019116453; URL: https://www.crd.york.ac.uk/prospero/display_record.php?RecordID=116453).

### Literature search

A systematic literature search was conducted in Embase, Medline Epub (Ovid), PsychInfo (Ovid), Web of Science and Google Scholar in May 2021 to identify relevant studies published after January 1980. The following keywords and their synonyms were included in the search: “parenting”, “stress”, “self-efficacy”, “competence”, “functioning”, “determinant”, “predictor”, “poverty” and “education”. The search strategy was adapted for each database accordingly. Relevant reviews and included articles were hand-searched for additional eligible studies. The search was developed and performed together with experienced information specialists from Erasmus Medical Centre. The complete search strategies are described in the supplementary material (**Supplementary material S1**).

### Inclusion and exclusion criteria

Included studies: (1) were articles published in peer-reviewed journals; (2) were available in English; (3) included parents with child(ren) (age range between 0 and 12 years) in the general population; in relation to this inclusion criterion, comparison studies where a specific populations was compared to a “general population sample” (e.g., parenting children with diabetes vs parenting children without diabetes) were only included when the results were separately reported for the comparison group. Only the findings in this comparison group were included in this review (4) reported the total score of parenting stress index (PSI) or version hereof (e.g., the PSI short form) as parenting stress measurement [20, 23]. This fourth inclusion criteria ensured that the studies included in our systematic review were comparable. In addition, the well-established psychometric properties of the PSI underline this as an appropriate measurement to assess parenting stress; and (5) reported the association between at least one possible factor and parenting stress; parenting stress was reported as the outcome or mediator. Accordingly, studies were excluded if they were performed among parents at risk (e.g., parents/child with certain diseases or impairments). Studies using other parenting stress measurements or used subscales of PSI only were excluded. Qualitative studies, reviews, thesis, conference papers, and book chapters, as well as non-English articles were also excluded. Lastly, intervention studies were excluded, as it is difficult to determine the association of a specific factor with parenting stress in these multifaceted interventions.

### Selection process

All references were exported and managed using Endnote X9. Title/abstract screening was performed by two reviewers (YF&MB/JL/DW) independently based on the inclusion criteria. Relevant articles were retrieved for full-text reading and further review by two reviewers (YF& MB/JL). Disagreements were discussed by the two authors (YF&MB/JL) until they were in agreement. Remaining disagreements were discussed with a third author (DW/AG) until consensus was reached.

### Data extraction

Data from individual studies were extracted and organized using an extraction form by one reviewer (YF) and then verified by another reviewer (MB/JL). The extracted information included: first author, year of publication, study country, study design, population and characteristics (i.e., sample size, the mean age of participants, percentage of daughters, mean age of children), follow-up period or measurement moments (if applicable), factors studied, and the reported associations between the studied factors and parenting stress.

From cross‐sectional studies, the reported association between the factors and parenting stress at the same time point was extracted. From longitudinal studies, the association between the factors at baseline and parenting stress at the final measurement was extracted. We considered quantitative measures of association reported in the studies. The results from the multivariable associations were included whenever possible, otherwise, the univariate results were used. The associations between the studied factors and parenting stress were represented with “ + ” for a significant positive association, “ − ” for a significant negative association, and “0” for a null association.

### Quality assessment

Two reviewers (YF and JL) assessed the quality of included articles independently using the Standard Quality Assessment Criteria for Evaluating Primary Research Papers from a Variety of Fields (QualSyst) [29]. The QualSyst is a 14-item tool that allows for methodological and bias assessment in quantitative and qualitative studies with varying study designs. Three items, namely item 5 (random allocation), 6 (blinding of investigators), and 7 (blinding of subjects), were removed from the QualSyst due to the observational design of the included studies. A score was given to each item to indicate whether the study fulfilled a criterion (0 = no, 1 = partially, and 2 = yes). Scores of the 11 items were added up to create a total score. The total sum score was then converted into a percentage score (i.e., study total sum score divided by the total possible score of 22) and rated as “excellent” (scores of > 80%), “good” (70%–79%), adequate (55%—69%) and “low” (< 55%) [7, 30]. Disagreements were resolved via discussion until consensus was reached. Articles rated as low quality were excluded from further analysis.

### Data synthesis

A non-quantitative synthesis was performed to summarize the evidence for an association of a factor with parenting stress. Following the parent–child relationship model [20]), three groups of factors were used: parent, child, and situational [31]. Reports of similar factors were combined as one. The factors in each group were further divided into subgroups. Where factors fit into multiple categories, they were coded to the most proximal level.

The level of evidence for each factor was summarized based on the method used in previous reviews [7, 32–34]. The number of studies that reported the association of a specific factor with parenting stress was divided by the total number of studies that examined that factor. An association between a factor and parenting stress that was reported by 0–33%, 34–59% and 60–100% of individual studies, was represented using the labels: ‘0’ for no association, ‘?’ for a potential association, ‘+’ for a positive association and ‘−’ for a negative association. Double signs (i.e., ‘00’, ‘??’, ‘++’ and ‘−−’) were given if the association between a factor and parenting stress was reported by four or more studies (Table 1).

**Table 1:** Rules for data synthesis, based on Mazarello Paes et al, 2015 [32]

Number of studies(N)	Evidence supported by (n/N)*	Strength of evidence	Symbols
<4	0–33%	No association	0
	33–59%	Indeterminate/possible association	?
	60–100%	Significant positive/negative association	–/+
≥ 4	0–33%	No association	00
	33–59%	Indeterminate/possible association	??
	60–100%	Significant positive/negative association	–/++

*n represents the number of studies reporting a significant association, *N* represents the total number of studies investigating the association

## Results

### Search results

**Fig. 2:**
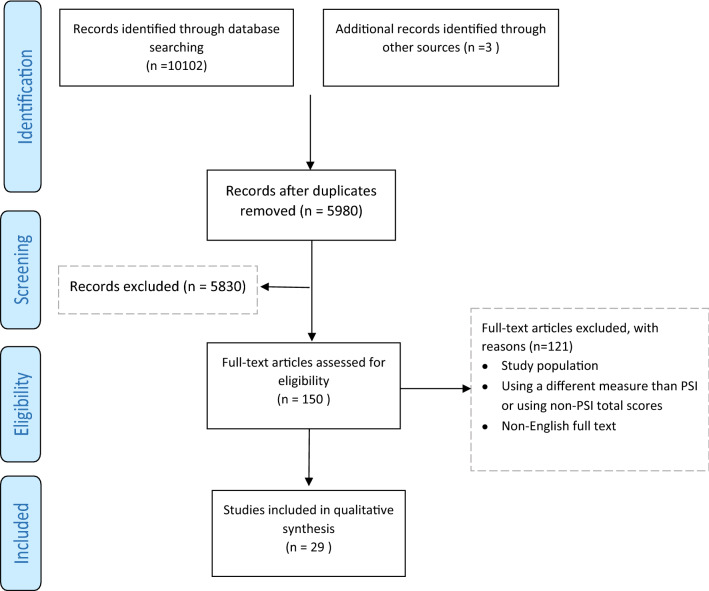
PRISMA flow diagram of study selection

The flow diagram of the included articles is presented in Fig. 2. The search resulted in 5980 articles after deduplication, which were screened on title and abstract. The full-text screening of the remaining 150 articles resulted in 29 articles that were eligible for inclusion in this systematic review.

### Characteristics of the included studies

Table 2 presents the overview of the characteristics of the studies included in this review. Detailed information including the first author, year of publication, study design, and methodology can be found in Supplementary Table 1. Overall, 20 (69.0%) studies used a cross-sectional design and 9 (31.0%) used a longitudinal design. Most studies (*n* = 22, 79.3%) were published after 2010, seven studies (24.1%) were published before 2010. Nearly half of the studies (*n* = 11, 37.9%) were conducted in North America, in Asia (*n* = 8, 27.6%), and in Europe (*n* = 6, 20.7%). The remaining studies were conducted in South America (*n* = 2, 6.9%), Australia (*n* = 1, 3.4%) and Africa (*n* = 1, 3.4%), respectively.

**Table 2:** Characteristics of the studies included in the systematic review (*N* = 29)

Characteristics	*N*	Percentage (%)
Study design		
Cross-sectional	20	69.0
Longitudinal	9	31.0
Location		
North America	11	37.9
South America	2	6.9
Europe	6	20.7
Asia	8	27.6
Australia	1	3.4
Africa	1	3.4
Year of publication		
< 2010	7	24.1
2010–2020	16	55.2
> = 2020	6	20.7
Study population		
Mothers only	15	51.7
Fathers only	1	3.4
Both^a^	8	27.6
Parents^b^	5	17.2
Number of participants		
< 100	7	24.1
100–299	10	34.5
300–999	7	24.1
> 1000	5	17.2
Age period^c^		
Infant (0-1y)	4	13.8
Pre-school age (1–4y)	15	51.7
School age (4–12y)	9	31.0
Not specific^d^	1	3.4
Measurement used		
Parenting stress index-standard form	4	13.8
Parenting Stress Index-short form	20	69.0
Translated or reduced version of PSI/ PSI-SF	5	17.2

^a^Parents were included in the study and subgroup analyses were performed to analyze associations for mothers and fathers separately

^b^Parents were included in the study and no subgroup analysis for mothers and fathers was performed

^c^Based on the mean age of children at the final measurement

^d^Delvecchio et al. [39] only reported range of the children (0–13 years), and therefore, was categorized into the not specific age group

Fifteen (51.7%) studies included only mothers, one (3.4%) included only fathers. Thirteen (44.8%) studies included a sample of both mothers and fathers (i.e., parents irrespective of gender), among which eight studies have examined the potential gender difference in mothers and fathers. Further, children's ages ranged from 0 to 12 years old, 13.8% (*n* = 4) included infants-only (0–1 years), 51.7% (*n* = 15) included preschool-aged children-only (1–4 years), 31.0% (*n* = 9) included school-age children-only (4–12 years), and 3.4% (*n* = 1) included both preschool and school-aged children.

### Quality of the included studies

The overall scores from the QualSyst checklist ranged from 59.1% to 100%, with a mean score of 81.8% ± 11.1%. Of the 29 included studies, sixteen (55.2%) were of excellent quality, nine (31.0%) were of good quality, and four (13.8%) were of adequate quality. No articles were of low quality; thus, no studies were excluded from analysis (Supplementary Table 2).

### Factors associated with parenting stress

**Fig. 3:**
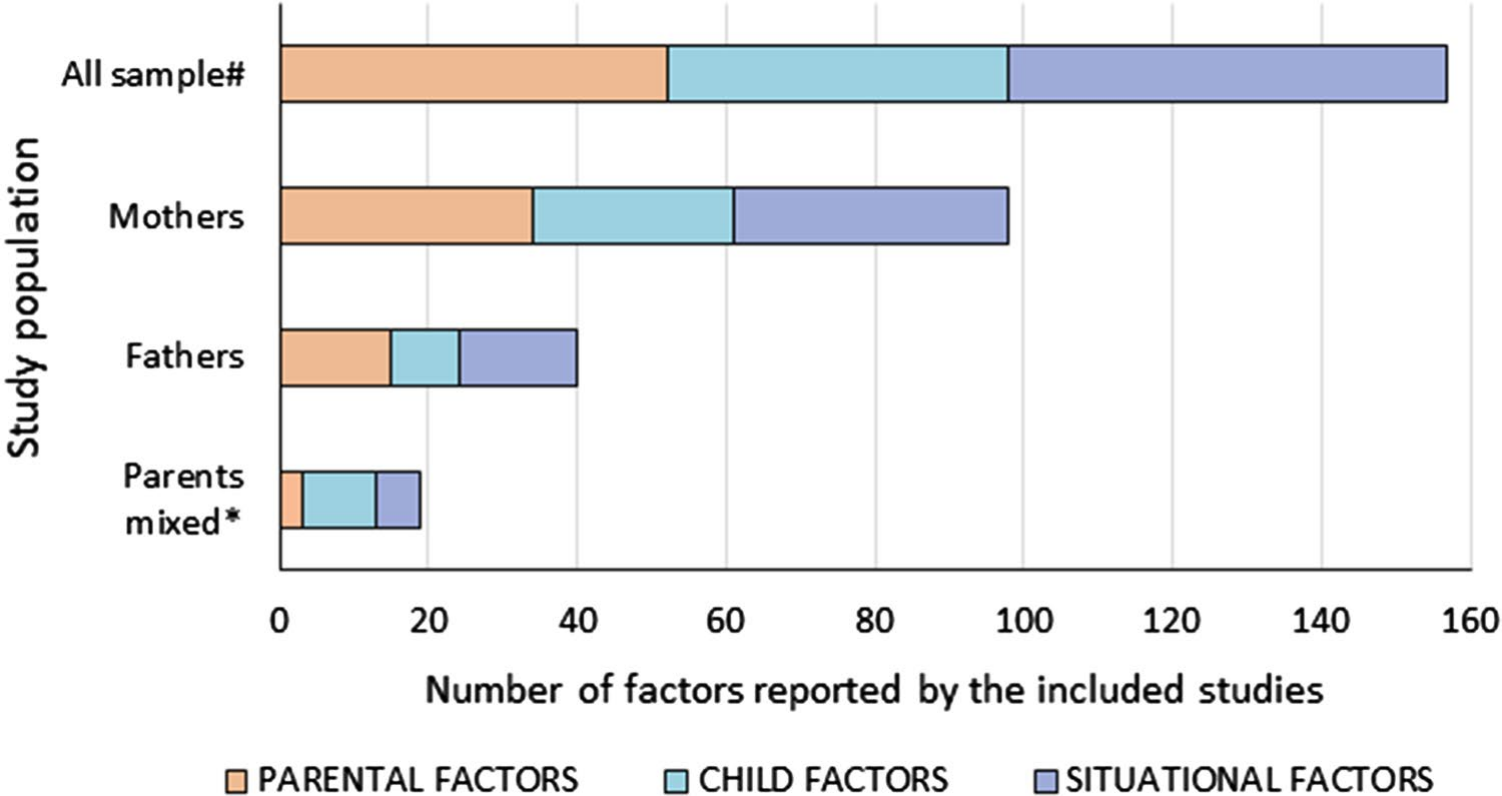
Distribution of the parental, child, and situational factors reported to be associated with parenting stress among parents of children aged 0–12 years old in the general population. Parents mixed*: parents were included in the study and no subgroup analysis for mothers and fathers were performed; ^#^ a factor was counted multiple times if presented in multiple groups

In total 111 unique factors were reported in the included studies. Hereof, 89.2% (*n* = 99) of the factors were reported by one or two studies, 7.2% (*n* = 8) of the factors were reported by three studies, and 3.6% (*n* = 4) of the factors were reported by four or more studies. The most frequently studied factors were parental factors, followed by situational factors and child factors (Fig. 3). In the current study, we present the results for mothers, fathers, and parents (i.e., irrespective of gender) separately. Strong evidence for an association between a certain factor and parenting stress is presented first in each paragraph (i.e., those factors reported upon in four or more than four studies) and less evidence second (i.e., factors reported upon in less than four studies) (see also Table 3).

**Table 3:**
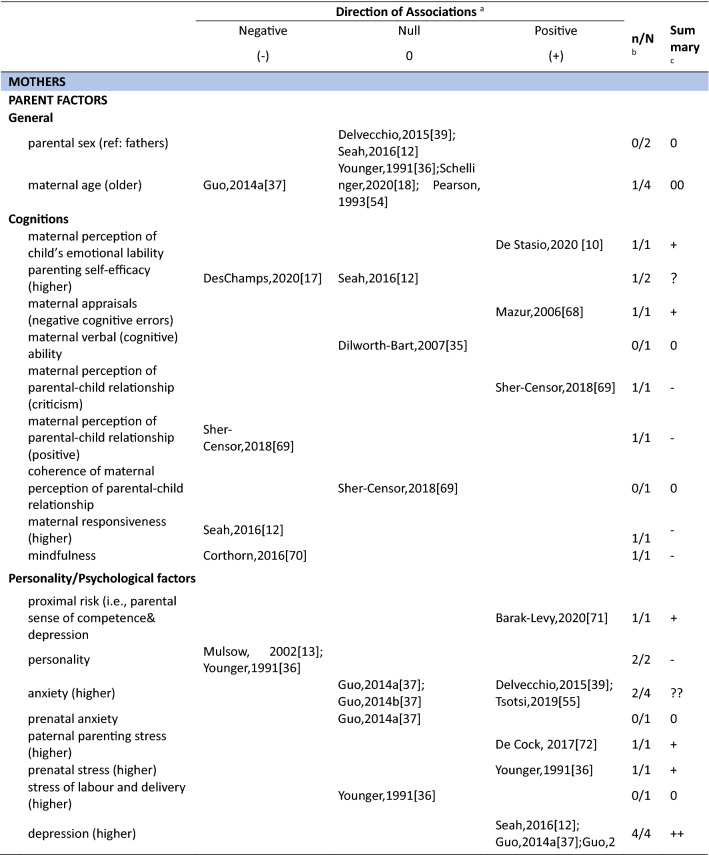

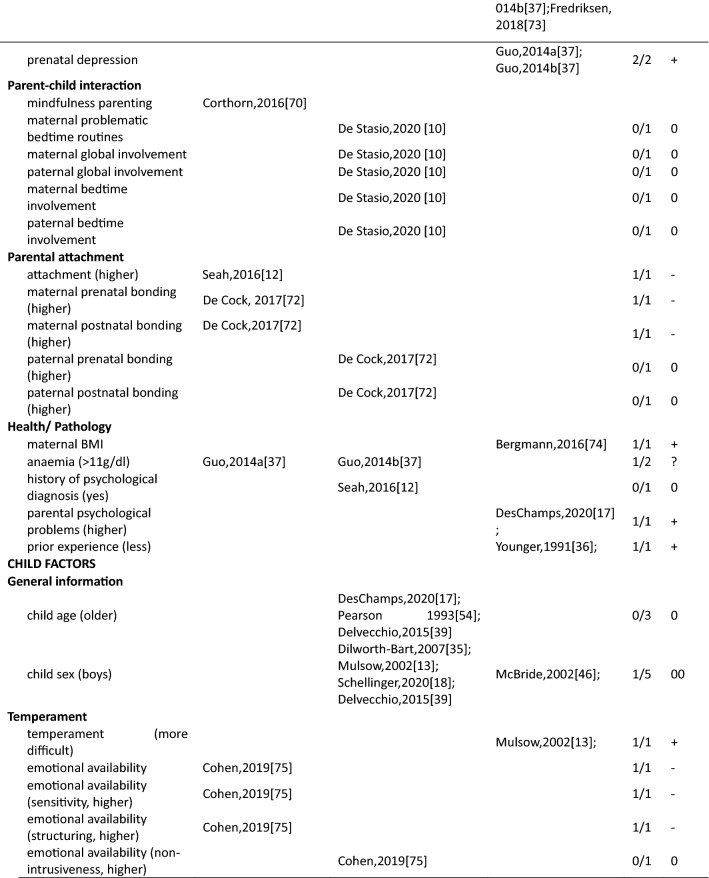

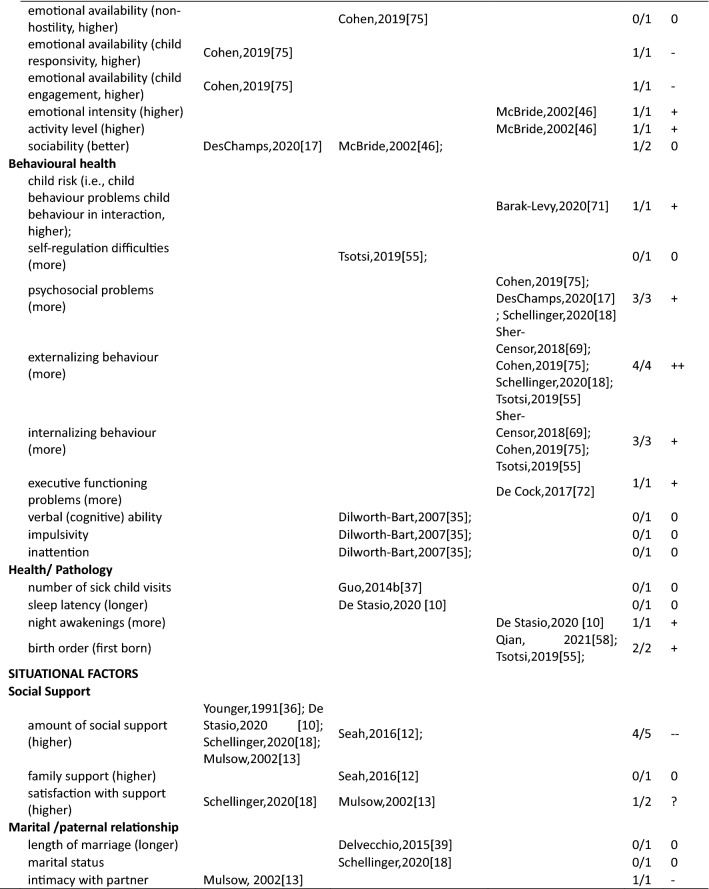

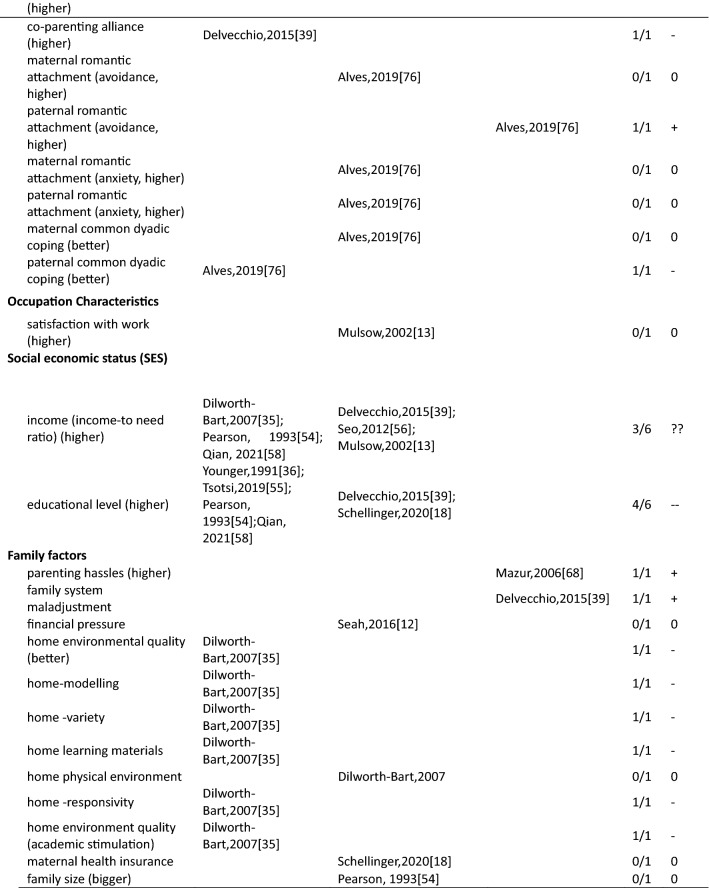

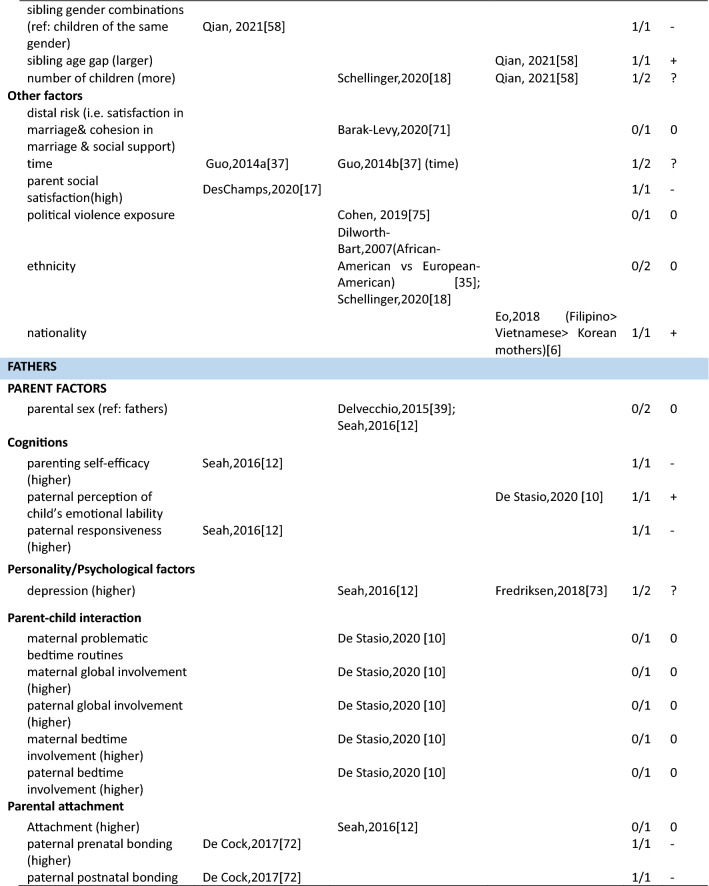

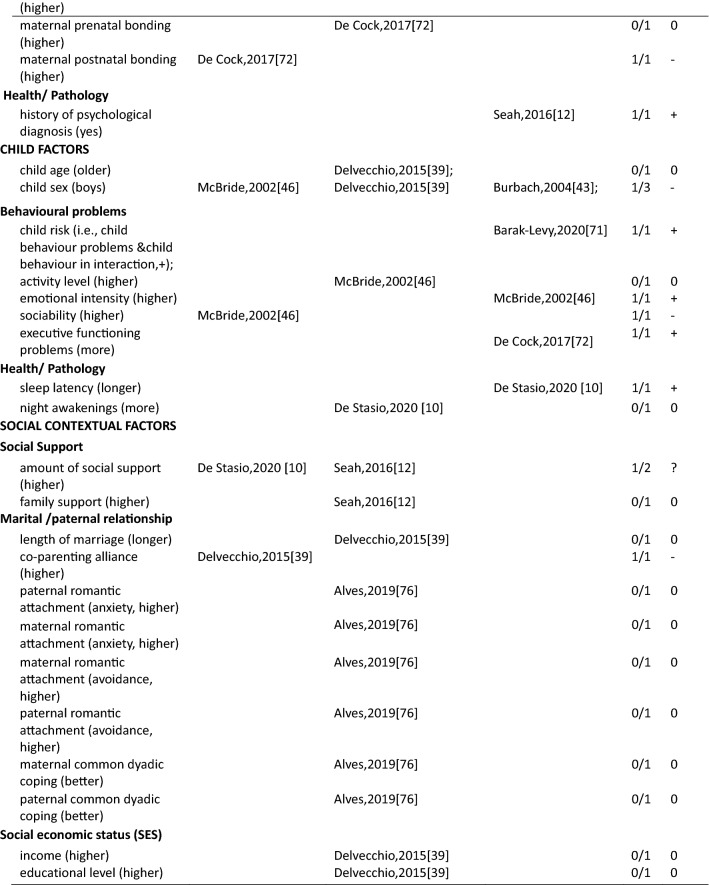

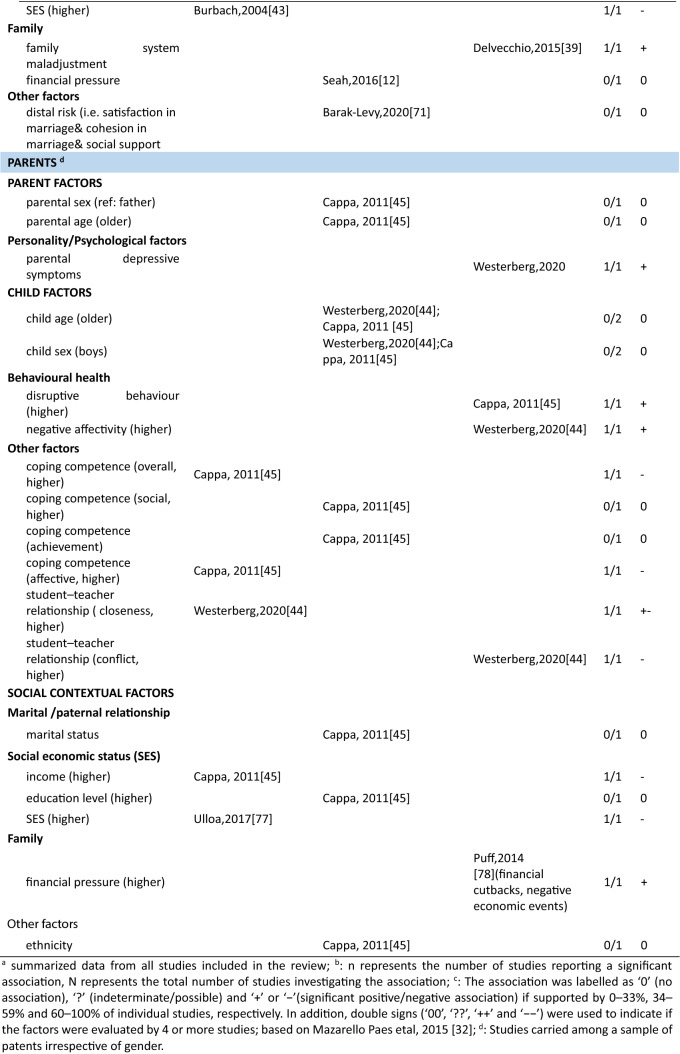
Associations between factors and parenting stress in the general population of parents with children between 0 and 12 years, reported by studies included in this review (*n* = 29)

#### Factors associated with parenting stress: Parent factors

##### Mothers

In total, thirty-four parent factors were reported in 18 studies with a sample of mothers. Out of the 34 studied factors, most (*n* = 31, 91.2%) were only reported in one or two studies. The three most often studied factors were maternal depression, anxiety and age. Studies among mothers showed a significant association between higher maternal depression and more parenting stress (4/4). There was inconsistent evidence for a positive association between maternal anxiety and parenting stress (2/4). Four studies found a null association between maternal age and parenting stress (3/4). The remainder of the factors were studied only once or twice. Among these, eight positive, nine negative, three inconsistent and eleven null associations with parenting stress were observed (see Table 3 for details).

##### Fathers

Fifteen parent factors were reported in six studies with a sample of fathers. All these factors were studied in one or two studies. For the 15 factors, there was some evidence for an association between fathers’ perception of child’s emotional lability (1/1), fathers’ history of psychological diagnosis (1/1) and more parenting stress. Higher parenting self-efficacy (1/1) and paternal bonding (1/1) were reported to be negatively associated with parenting stress. There was inconsistent evidence for an association between depression (1/2) and parenting stress in fathers. Other parent factors including attachment (0/1), maternal prenatal bonding (0/1), bedtime routines, and global/bedtime involvement were not related to parenting stress.

##### Parents

Three parent factors were identified in two the studies with samples of parents (i.e., irrespective of gender). Factors were studied in one or two studies. There was no evidence for associations of parent sex (0/1), age (0/1) and parenting stress. However, parental depressive symptoms were associated with higher levels of parenting stress (1/1).

#### Factors associated with parenting stress: Child factors

##### Mothers

Twenty-seven child factors were identified in 14 studies with a sample of mothers, of which 22(81.5%) were reported in one or two studies. Maternal parenting stress tended to increase with child’s psychosocial problems (3/3), externalizing (4/4) and internalizing behavioral problems (3/3). There was no evidence that maternal parenting stress differs by child age (0/3) or sex (1/5). Among other child factors, seven positive, five negative and ten null associations were reported.

##### Fathers

Nine child factors were identified in six studies with a sample of fathers, of which eight were reported in one or two studies. There was no evidence that fathers parenting a boy would experience higher parenting stress than fathers parenting a girl (1/3). Regarding the other eight child factors, four positive [child overall risk (1/1), emotional intensity (1/1), executive functioning problems (1/1), prolonged sleep latency (1/1)], one negative [sociability (1/1)] and three null [child age (0/1), activity level (0/1), and night awakenings (0/1)] associations were reported.

##### Parents

Ten child factors were identified in two studies with a sample of parents. These were mainly reported by one study only. There was some evidence that parents of children with more behavioral problems (disruptive behavior, 1/1; negative affect, 1/1) have higher parenting stress. Better student–teacher relationship [i.e., higher closeness (1/1) and lower conflict (1/1)] was associated with higher parenting stress. There was no evidence for associations between child age (0/2) and sex (0/2). One study focused on children’s coping competence and parenting stress and reported mixed associations.

#### Factors associated with parenting stress: Situational factors

##### Mothers

Thirty-seven situational factors were identified in 14 studies with a sample of mothers, of which 34 (91.8%) were reported by one or two studies. There was evidence for associations between a higher amount of social support (4/5), higher educational level (4/6) and lower levels of parenting stress. There was inconsistent evidence for associations between family income (3/6) and parenting stress. Two studies investigated the association between satisfaction toward support and parenting stress (1/2) [11, 13]. Two studies investigated the role of ethnic background and reported null associations (0/2) [11, 35]. One study reported a null association between occupational satisfaction and parenting stress [13]. Regarding factors related to marital/ paternal relationship, there was some evidence of an association between higher intimacy with partner (1/1), higher co-parenting alliance (1/1), lower paternal romantic avoidance (1/1), higher paternal common dyadic coping (1/1) and lower parenting stress. There was no evidence of an association between parenting stress and other marital factors. One study investigated the association between home environmental quality and parenting stress, and reported inconsistent results [35].

##### Fathers

Sixteen situational factors were identified in six studies with a sample of fathers. There was some evidence of an inconsistent association between the amount of social support (1/2) and parenting stress. Evidence for factors related to marital/paternal relationship, family, and social economic status (SES) was reported by single studies; two negative, one positive and twelve null associations with parenting stress were found.

##### Parents

Six situational factors were identified in three studies with a sample of parents. There was some evidence for an association between lower income (1/1) and higher parenting stress. Factors including marital status (0/1), paternal educational level (0/1), and ethnicity (0/1) were not associated with parenting stress. Other factors including lower family SES (1/1), and higher family financial pressure (1/1) were associated with higher parenting stress. However, these factors were reported by single studies only.

## Discussion

This review aimed to provide an overview of the available literature evaluating factors associated with parenting stress among parents of children aged 0–12 years from the general population. A large number of factors in the parent, child, and situational domains were identified across the 29 studies included. Overall, the majority of the factors was studied only once or twice, predominantly in samples of mothers and studies applying a cross-sectional design. There was evidence that several factors were associated with parenting stress.

In our discussion below, we mainly focused on the factors that have been reported in at least four studies in this review and then drew on previous research to suggest the potential difference in parenting stress among mothers and fathers. Next, we suggest directions for future research. Finally, we discuss the methodological considerations of the current study.

### Factors associated with parenting stress

In general, most of the factors were in line with Abidin's model [2, 20]. Parental factors are the most extensively studied factors in the studies included, and the included studies specifically focused on parents’ personality/psychological status. Situational factors seemed to have received relatively little attention. And there is variability among situational factors studied. Variables including social support, marital relationship, family environment, and socioeconomic status were studied often, while other variables, such as culture and employment experience, were less studied. Finally, studies on the child domain reported on factors, such as child problem behaviors and difficult temperament, related to perceived higher parenting burden.

#### Parent factors and parenting stress

There is evidence for a null association between maternal age and parenting stress in mothers. Two out of the three studies that reported a null association had included a sample of mothers with a wide age range (17–40 years [36] and 16–40 years [11]). While, the third study included a sample of mothers with a smaller age range/ on average 29 years found a negative association between maternal age and levels of parenting stress [37]. The association might be non-linear. For instance, hypothetically very young and older mothers experience higher stress levels [36, 38].

According to Abidin, parental personality and psychopathology play an important role in the construction of parenting stress [2, 20]. In line herewith, this systematic review included studies reported that anxiety and depression were risk factors for higher parenting stress levels. These undesirable psychological statuses may undermine parents’ ability to initiate and maintain positive affective interactions with their children and other family members; moreover, these parents are also likely to display dysfunctional parenting [39]. As a result, they may find parenting more demanding [40, 41]. Previous studies showed that mothers who are anxious or depressed might need extra support in managing the stressor in their parenthood [42]. Therefore, our findings underline the need for health professionals to support parents when needed.

#### Child factors and parenting stress

There is no evidence that parents who have a child that is a boy compared to parents that have a child who is a girl differ in their perception of parenting stress in this systematic review. Most studies included in this review indicate that child gender is not a relevant factor for parenting stress [11, 13, 35, 39, 43–45]. Differences were noted in one study, where parents of boys reported higher total parenting stress than parents of girls [46]. Although the general conclusion drawn from the included studies is that child gender is not associated with parenting stress. Child gender can still function as moderator in the association between certain factors and parenting stress. For instance, McBride et al. reported that the association between child temperament and parenting stress differed on the basis of child and parent gender; the difference in stress associated with low versus high levels of emotional intensity exists mainly for mothers of boys and fathers of girls [46]. In a comprehensive review, Leaper elaborated how child gender could affect parenting and the other way around [47]. Therefore, it is likely that parent and child gender interact with each other also in relation to parenting stress. Potential pathways should be explored in future research.

Children’s psychosocial problems, externalizing and internalizing problems are frequently associated with parenting stress in longitudinal and cross-sectional studies [14, 48]. Children with these psychosocial problems have been characterized as being more likely to have a difficult temperament [49] and being demanding [50]. Parenting for children with psychosocial problems could therefore also be more challenging, and especially when the demands of raising their children exceed available resources, parents could experience increased parenting stress as predicted by Abidin's model [2, 20]. Additionally, the associations between child behavioral problems and parenting stress could be reciprocal [51]. Our results emphasize the importance of early support for parents parenting children with psychological problems.

#### Situational factors and parenting stress

Based on this systematic review, the findings from included studied suggest that higher levels of social support are related to less parenting stress. Social support, which refers to the social connections or resources provided by others, is a well-recognized resource for parents to facilitate coping with stressful or difficult circumstances [16]. Social support may affect parenting stress through its direct or indirect impact on other factors [2, 20]. For instance, compared to their peers, parents perceived more social support are less depressed, less restricted in their role of parenting, have higher parenting self-efficacy, and would be better able to cope with a demanding child [7, 52, 53]. Most studies on social support included in this review had focused on the amount of social support and perceived social support [10–13, 37]. Only a few studies focused on the source of support [12], and parents’ satisfaction with the support [11, 13]. However, the actual availability of social support, the source, content, quality and parents’ satisfaction toward the support could also be important components of social support. More studies examining these factors could provide a better understanding of how different contexts of social support could affect parenting stress. These studies could also be informative for interventions aiming at reducing parenting stress through boosting social support.

In addition to the amount of social support, the economic status of the mothers might also be related to maternal parenting stress. The SES was by most studies measured via education and/or income separately [11, 13, 35, 36, 39, 54–56]. Together, these studies suggested evidence for an association between having a higher maternal educational level and lower parenting stress; and inconsistent evidence for an association between income and maternal parenting stress. Mechanisms underline the association between SES and parenting stress are myriad and may involve factors including an undesirable home environment and available coping resources [19, 57]. Previous studies have found that compared to less-educated parents, highly educated parents might have more parenting knowledge and social resources, and higher abilities to cope with the anxieties and stress in their parenting roles [57, 58]. As for family income, studies included have used different income indicators. For instance, Dilworth-Bart [35] and Mulsow [13] have used ‘income-to-ratio’, in which family size was also considered. As stated above, the association between SES and parenting stress may be complex and may not be explained by simple regression analysis. Therefore, we recommend future studies to further investigate the role of SES in parenting stress.

### Mothers, fathers and parenting stress

Mothers and fathers may perceive their role as a parent differently. Several explanations have been posited for this disparity, including differential perception, coping and experience of stress [59, 60], and differences in the willingness of men and women with regard to help-seeking [60]. Moreover, compared to men, women might be more vulnerable to stress raised from problems like marital conflict and might also have lower coping abilities toward these problems [59, 60]. Eight studies included in this systematic review were performed in mixed samples of mothers and fathers (Supplementary Table 3). Among these studies, two studies examined the gender difference with mixed evidence [12, 39]. Among the 38 factors reported in both mothers and fathers, most are in similar directions for both fathers and mothers. Some factors seem to affect mothers and fathers differently when it comes to parenting stress. For example, attachment was reported to be associated with maternal parenting stress only [12], while child sociability was associated with paternal parenting stress [46]. However, there is not enough research to comment on the strength or importance of these associations, as they were reported only in one or two studies. Fathers are generally underrepresented in parenting-related studies [7]. In the included studies of this systematic review, one study had focused only on samples of fathers [43]. The culture of parenthood has been changing over the past few decades, and there is an increase in caring fatherhood [61]. In future studies, representative samples of mothers and fathers are recommended.

### Methodological considerations

The strengths of the systematic review include the use of a broad set of search terms for parenting stress (i.e., parenting self-efficacy and parenting sense of competence) to identify published papers on this topic. Another strength is the inclusion of high-quality studies, according to the criteria by Kmet, Cook [29]. This review adds to the existing literature by presenting a data synthesis of available literature for the associations among mothers and fathers. Moreover, parenting stress in the general population was evaluated, which is useful for intervention development and support provided by community health professionals.

However, several limitations should be addressed as well. First, this review focused on parenting stress measured by the PSI only. Although there are also other parenting stress measures (e.g., the parental stress scale [62]), PSI remains to be the most frequently and widely used parenting stress measure since it was first proposed in 1983 [14, 22, 23]. Including studies that use a similar measure of parenting stress, increased the comparability of the findings across studies. However, there is similarity in the factors studied and the PSI measure. For instance, items measuring parent distress are comparable to the measures of parent depression, and items measuring difficult child in PSI are similar to measures of child temperament or problematic behavioral characteristics. Therefore, multi-collinearity could be possible. Moreover, this might have also lead to an overestimation of the strength of certain associations in individual studies in our review.

In the current review, the association between factors and the PSI total score was evaluated. Six studies in our review reported the association between factors and both the PSI total, the parent domain, and the child domain (see  Supplementary Table 4). We explored whether the associations reported between factors and the total score, the parent and the child domain were comparable. However, given the few number of studies, findings were inconsistent. Future (review) studies could focus on a smaller age group (e.g., 2–4 years) to study in depth the factors associated with parenting stress. In such studies, it would be feasible to include several instruments to assess parenting stress, as well as domain-specific outcomes, to identify potential intervention opportunities.

In addition, since PSI was used with parents whose children are under 12 years, we were unable to study factors associated with parenting stress among parents of adolescents. Second, the framework of Abidin was used to group factors and present findings. The model was developed in the 1980s, since then there have been significant societal changes for instance, in family structure, caregiving roles and a host of factors emerged. Although this framework captures the most well-supported factors related to our current understanding of parenting stress, some factors (e.g., mindfulness) that might also fit into the domains of parent, child, and situational factors that might be added to Abidin’s model. This systematic review does not yet consistently support these new factors. Furthermore, the initial model assumes that all factors to have equal potential with regard to total stress load, and the interactive effects of factors were not considered [20]. Future research investigating the pathways over time could help further expand the model. Third, causalities cannot be ascertained as most of the studies included in this review followed a cross-sectional design. In addition, the frequency of the factors reported was used to determine the strength of the evidence. Limitations to this methodology have been reported (e.g., most reported factor might not be the most important one) [63]. However, this method is transparent in its processes and easily auditable and has been widely used in systematic reviews on correlates studies [33, 34, 64, 65]. And finally, publication bias cannot be ruled out as only peer-reviewed papers in the English language were included.

### Implications and future studies

The findings from this systematic review provide valuable information for health care practice. Certain factors, namely maternal depression, children’s overall problems, externalizing, and internalizing behaviors, were associated with higher levels of parenting stress in parents. This information can help health care professionals to better detect parents and families experiencing parenting stress. In addition, higher levels of social support were associated with lower parenting stress in mothers. While working to reduce stress, health professionals could also try to maximize parents’ resources to handle. Increasing social support could be considered as a preventive strategy or support strategy in supporting parents [66, 67].

Several recommendations for future research can be formulated. Overall, most of the associations presented in the selected studies were based on few data points. And some of the included studies were rated as adequate quality, as a consequence of the study designs and analysis: small sample size and lack or insufficient control of confounders. So first, future studies should apply high-quality prospective study designs to examine how parent, child, and situational factors might affect parenting stress over time. Second, more representative samples should be studied. Specifically with regard to parent gender, only 12.5% (2634 fathers vs. 18,436 mothers) of the participants in the included studies were fathers. As mentioned above, there is an increment of caring fatherhood in the past decades, and previous studies suggest that the patterns and predictors of parenting stress could differ between male and female caregivers [1, 18]. Third, although our study included samples with children aged 12 years or younger, many studies of parenting stress in the developmental literature have focused on young children (< 6 years). While the experience of parenting stress may differ across child developmental stages. For instance, parents with very young children or adolescents might perceive more stress [24]. Therefore, future studies need to distinguish parenting stress across different age ranges. Fourth, this review identified several potential factors associated with parenting stress; however, the majority of factors were only investigated by one/two studies. Also, several key variables, such as cultural factors, family stress, working experience, and stressful life events, were not considered in the included studies. Moreover, as discussed above, a set of new factors emerged with the development of the society. These are promising variables for future studies to better understand their influence on parenting stress. In addition, future studies should continue to investigate the pathways (mediations) and interactions (moderations) between factors in the association with parenting stress.

## Conclusion

The factors that underlie parenting stress are multifaceted and complex. This systematic review provides a detailed overview of studies into parent, child, and situational factors associated with parenting stress. Findings indicate that some factors (e.g., parental depression and amount of social support) might be modifiable through tailored support by health care professionals. More research, especially longitudinal studies with representative samples of men and women are needed to study parent, child, and situational factors associated with parenting stress to inform the development of prevention and intervention strategies.

### Supplementary Information

Below is the link to the electronic supplementary material.

Supplementary file1 (DOCX 13 KB)

Supplementary file2 (XLSX 34 KB)

## Abbreviations

SD: Standard deviation
SES: Social Economic Status
QualSyst: Standard Quality Assessment Criteria for Evaluating Primary Research Papers from a Variety of Fields
PSI: Parenting Stress Index
