# Supplementary material for: Parent, child, and situational factors associated with parenting stress: a systematic review
Source: Eur Child Adolesc Psychiatry. 2022 Jul 25;33(6):1687–705. doi: 10.1007/s00787-022-02027-1 (PMC11211171; doi:10.1007/s00787-022-02027-1)
Supplement: Supplementary file 1 — Supplementary file1 (DOCX 13 KB) [file 787_2022_2027_MOESM1_ESM.docx]

**Supplementary S1:** Search strategy

***Embase.com (Embase incl. Medline): 1732***

**(**'parental stress'/de OR 'parenting stress index'/de OR 'parenting stress index short form'/de OR (((parenting OR parental OR maternal OR paternal) NEAR/3 (concern* OR competen* OR hassl* OR empower* OR uncertain* OR dysfunction*)) OR ((parenting OR parental OR maternal OR paternal) *NEXT/2* (stress* OR efficacy))):ab,ti**) AND (**'social determinants of health'/de OR 'determinant'/de OR 'predictor variable'/de OR 'prediction'/de OR 'income group'/exp OR 'educational status'/exp OR 'poverty'/de OR 'household income'/exp OR (determinant* OR predictor* OR predictive OR prediction* OR ((education* OR income) NEAR/3 (status OR level OR group* OR family OR household OR background)) OR poverty OR ((low OR high OR middle OR moderate) NEXT/1 (income))):ab,ti**) NOT** ('Conference Abstract' OR 'Editorial')/it

***Medline Epub (Ovid): 1731***

**(**(((parenting OR parental OR maternal OR paternal) ADJ3 (concern* OR competen* OR hassl* OR empower* OR uncertain* OR dysfunction*)) OR ((parenting OR parental OR maternal OR paternal) ADJ2 (stress* OR efficacy))).ab,ti.**) AND (**Social Determinants of Health/ OR exp Forecasting/ OR "Income"/ OR exp "Educational Status"/ OR "Poverty"/ OR (determinant* OR predictor* OR predictive OR prediction* OR ((education* OR income) ADJ3 (status OR level OR group* OR family OR household OR background)) OR poverty OR ((low OR high OR middle OR moderate) ADJ1 (income))).ab,ti.**) NOT** (congresses OR editorial).pt.

***PsycInfo (Ovid): 2340***

**(**(((parenting OR parental OR maternal OR paternal) ADJ3 (concern* OR competen* OR hassl* OR empower* OR uncertain* OR dysfunction*)) OR ((parenting OR parental OR maternal OR paternal) ADJ2 (stress* OR efficacy))).ab,ti.**) AND (**exp Prediction/ OR exp "Income Level"/ OR exp "Educational Background"/ OR "Poverty"/ OR (determinant* OR predictor* OR predictive OR prediction* OR ((education* OR income) ADJ3 (status OR level OR group* OR family OR household OR background)) OR poverty OR ((low OR high OR middle OR moderate) ADJ1 (income))).ab,ti.**) NOT** (congresses OR editorial).pt.

***Web of Science: 2462***

**TS=((**(((parenting OR parental OR maternal OR paternal) NEAR/2 (concern* OR competen* OR hassl* OR empower* OR uncertain* OR dysfunction*)) OR ((parenting OR parental OR maternal OR paternal) *NEAR/1* (stress* OR efficacy)))**) AND (**(determinant* OR predictor* OR predictive OR prediction* OR ((education* OR income) NEAR/2 (status OR level OR group* OR family OR household OR background)) OR poverty OR ((low OR high OR middle OR moderate) NEAR/1 (income)))**)**) AND DT=(Article)

***Google Scholar: 200 (****top relevant references)*

**"**parenting|parental|maternal|paternal stress|concern|competence|hassle|empowerment|uncertainty|dysfunctioning|efficacy" determinant|determinants|predictor|predictive|prediction|poverty|income
